# Immune-cold NSCLC tumors harbor tumor-associated macrophages with elevated expression of immunoglobulin genes

**DOI:** 10.1007/s00262-026-04446-4

**Published:** 2026-06-06

**Authors:** Markus Haug, Henrik Sahlin Pettersen, Magne Børset, Arne Kildahl-Andersen, Sissel Gyrid Freim Wahl, Arnar Flatberg, Anders Tøndell

**Affiliations:** 1 Department of Thoracic Medicine, St. Olavs Hospital, Trondheim University Hospital, Postboks 3250 Sluppen, No 7006, Trondheim, Norway; 2 Department of Pathology, St. Olavs Hospital, Trondheim University Hospital, Trondheim, Norway; 3 Department of Immunology and Transfusion Medicine, St. Olavs Hospital, Trondheim University Hospital, Trondheim, Norway; 4 Department of Circulation and Medical Imaging, Norwegian University of Science and Technology (NTNU), Trondheim, Norway; 5 Department of Clinical and Molecular Medicine, Norwegian University of Science and Technology (NTNU), Trondheim, Norway; 6 Department of Infectious Diseases, St. Olavs Hospital, Trondheim University Hospital, Trondheim, Norway

**Keywords:** Non-small cell lung cancer, Immune response, Tumor-associated macrophages, Tumor microenvironment

## Abstract

In the non-small cell lung cancer (NSCLC) tumor microenvironment (TME), tumor cells mediate inhibitory signals to key immune cells, promoting an immunosuppressive environment that facilitates tumor immune evasion. CD8 + cytotoxic T lymphocytes are central mediators of antitumor immunity, and tumors can be classified by immunohistochemistry (IHC) as immune-hot or immune-cold based on the abundance of tumor-infiltrating lymphocytes (TILs). Although cancer immunotherapy can boost lymphocyte functions and improve progression-free survival in patients with advanced NSCLC, only a minority of patients experience a durable clinical benefit. Tumor-associated macrophages (TAMs) are key immunoregulatory cells in the NSCLC microenvironment and capable of generating potent immunosuppressive signals. However, their functional roles in immune-hot versus immune-cold tumors remain poorly understood. In this study, we compared the transcriptional programs of TAMs from NSCLC patients with immune-hot or immune-cold tumors. We classified 11 surgically resected NSCLC tumors as immune‑hot or immune‑cold based on quantitative immunohistochemistry of CD4⁺ and CD8⁺ tumor‑infiltrating lymphocytes. TAMs were isolated from tumor and adjacent healthy tissue by fluorescence-activated cell sorting (FACS), followed by bulk RNA sequencing and differential gene expression analysis. TAMs from immune‑cold tumors exhibited a striking upregulation of genes involved in immunoglobulin-mediated immune responses. Additionally, these TAMs demonstrated increased expression of genes involved in extracellular matrix organization, including matrix metalloproteinases and collagen‑associated genes, suggesting enhanced matrix remodeling activity. These findings highlight TAMs’ potential contribution to immunosuppression, stromal remodeling, and impaired lymphocyte infiltration. The TAM-mediated pathways identified here may represent actionable targets for future immunotherapeutic strategies aimed at reshaping the tumor microenvironment.

**Supplementary Information:**

The online version contains supplementary material available at 10.1007/s00262-026-04446-4.

## Introduction

Lung cancer remains the leading cause of cancer-related deaths worldwide [1]. Non-small cell lung cancer (NSCLC) accounts for the majority of cases, with surgery being the preferred treatment option for potentially curable disease. For patients who are not suitable for surgery or have locally spread disease, radiation therapy is an alternative. Unfortunately, around 40% of patients present with metastatic disease at diagnosis [2, 3]. These patients typically receive platinum doublet chemotherapy combined with immunotherapy, or immunotherapy only. While the implementation of cancer immunotherapy has significantly improved progression-free survival in advanced NSCLC [4], long-term follow-up data indicate that only around 20% of patients experience a durable treatment response [5, 6].

The tumor immune microenvironment (TME) comprises various immune cells capable of mounting an anti-tumor response, including cytotoxic T lymphocytes and macrophages. In NSCLC, the TME is predominantly an immunosuppressive environment in which tumor cells mediate inhibitory signals to key immune cells, thereby facilitating tumor immune evasion [7, 8]. Immunotherapy for NSCLC aims to overcome this cancer-induced immunosuppression using monoclonal antibodies that target immune checkpoints such as the PD-1/PD-L1 pathway. This pathway is one of several immunosuppressive mechanisms exploited by NSCLC cells to escape immune surveillance and promote tumor progression [3].

Macrophages are phagocytic cells that capture antigens and present them on major histocompatibility complex (MHC) molecules, and express costimulatory molecules that interact with T and B cells. They are also a major source of diverse pro- and anti-inflammatory cytokines, which orchestrate diverse immune functions [9]. Alveolar macrophages serve as first-line defenders of alveoli, airways, vasculature and the lung interstitium [10]. In maintaining tissue homeostasis, alveolar macrophages mainly confer immunosuppressive signals [10]. Macrophages are now recognized for their high degree of plasticity, and a multitude of activation states exist with diverse functional capabilities [11, 12]. Within the lung TME, tumor-associated macrophages (TAMs) constitute the dominating immune cell population [11, 13, 14]. While TAMs have the potential to kill and remove tumor cells by phagocytosis, mediate antibody-dependent cellular cytotoxicity, and activate innate or adaptive lymphoid cells [15], they frequently generate strong immunosuppressive signals in the TME [15]. Elevated densities of TAMs are correlated with reduced survival and poor prognosis in various malignancies [16, 17].

Tumors can be classified as immune-hot or immune-cold based on the number of tumor-infiltrating lymphocytes (TILs) determined by immunohistochemistry (IHC) analyses [18]. This classification provides prognostic information as immune-hot tumors are associated with better long-term survival and a more favorable response to treatment [16, 19]. Immune-hot tumors are defined by high concentrations of infiltrating CD4 + and CD8 + T cells. While elevated TIL levels typically indicate an active anti-tumor immune response, local immunosuppressive signals can redirect these T cells toward functional impairment or exhaustion [20]. Alternatively, TILs may be excluded from the TME due to physical barriers or an immunosuppressive environment that limits recruitment [19]. The functional role of TAMs in immune-hot versus immune-cold NSCLC environments remains poorly understood, and it remains unclear whether discrete transcriptional programs distinguish TAMs found in these environments.

Previously, we identified highly differentially expressed genes (DEGs) in TAMs, CD4 + , and CD8 + T cells purified from the TME of NSCLC tumors compared to their counterparts in adjacent healthy lung tissue [21]. The primary aim of the present study was to identify gene programs preferentially activated in TAMs from immune-hot tumors compared to those from immune-cold tumors. We classified tumors from 11 NSCLC patients as immune-hot or immune-cold based on IHC and isolated TAMs from the tumors of these patients. Transcriptomic profiles of TAMs from immune-hot and -cold tumors were analyzed and compared. Results were further related to gene expression data from macrophages residing in adjacent healthy tissue.

## Materials and methods

### Study population

Patients with clinical stage I-III NSCLC scheduled for surgery were eligible for the study. Eleven patients were enrolled after providing written informed consent during the inclusion period from February 2017 through March 2018. The Regional Committee for Medical and Health Research Ethics approved the study (REC Central, Ref.nr.: 2010/1939).

Following video-assisted thoracoscopic surgery (VATS) with lobectomy, resected lung tissue was immediately stored on ice and brought to the laboratory. About ~ 1–3 cm^3^ of tumor and adjacent healthy lung tissue were harvested from the resected lobe. All samples used for immunohistochemistry (IHC), fluorescence-activated cell sorting (FACS), and RNA sequencing were generated from these tumor- and tissue specimens. See Table 1 for characteristics of the study population.

**Table 1:** Patient characteristics and concentration of tumor-infiltrating immune cells

Patient	Age	Sex	Smoker	Tumor	Stadium	Relapse	CD4 + T cells in tumor	CD8 + T cells in tumor	B cells in tumor	Macrophages in tumor	TILs in tumor	T cells in tumor	T cell high tumor
1	70–74	M	former	AC	IA3	No	746	349	170	382	1265	1095	Yes
2	70–74	M	current	AC	IVA	Yes	2463	2307	550	763	5320	4770	Yes
3	80–84	F	former	SCC	IIB	Yes	307	337	116	265	760	644	No
4	70–74	M	former	SCC	IIB	No	339	437	213	289	989	776	No
5	80–84	M	former	SCC	IB	No	82	38	12	109	132	120	No
6	70–74	M	former	AC	IIIB	Yes	692	495	72	94	1259	1187	Yes
7	70–74	M	former	AC	IIIA	Yes	956	339	537	207	1832	1295	Yes
8	75–79	F	former	SCC	IIA	Yes	240	233	57	221	530	473	No
9	55–59	F	current	AC	IIIA	No	720	330	287	146	1337	1050	Yes
10	75–79	F	current	SCC	IIB	No	539	363	339	249	1241	902	No
11	75–79	M	former	AC	IIB	No	776	698	336	275	1810	1474	Yes

Quantification of immune cells by immunohistochemistry in tumor tissue from patients with NSCLC. Immune cell concentrations are displayed as cells per mm^2^. Tumors are considered ‘T cell high’ if > 1000 T cells/mm^2^. AC: Adenocarcinoma; SCC: Squamous cell carcinoma; Stadium: histopathologic TNM Classification of Malignant Tumors (8th edition); Relapse: After a minimum of 12 months observation post-surgery

### Isolation of mononuclear cells from tumor and lung tissue

TAMs and macrophages from lung tissue were isolated as previously described [21, 22] using a procedure that was adapted from Quatromoni et al. [23]. In brief, the tissue was mechanically minced into 1–2 mm^3^ fragments by microdissection, then incubated with DNase, elastase and collagenase for enzymatic degradation, and the resulting solution was filtered through a 70 µm filter (BD Biosciences, San Jose, CA, US), to remove non-cellular debris and undigested material [23]. Subsequently, erythrocytes were lysed, and mononuclear immune cells were isolated by density gradient centrifugation, followed by several washing steps.

### Antibody staining and cell sorting

**Fig. 1:**
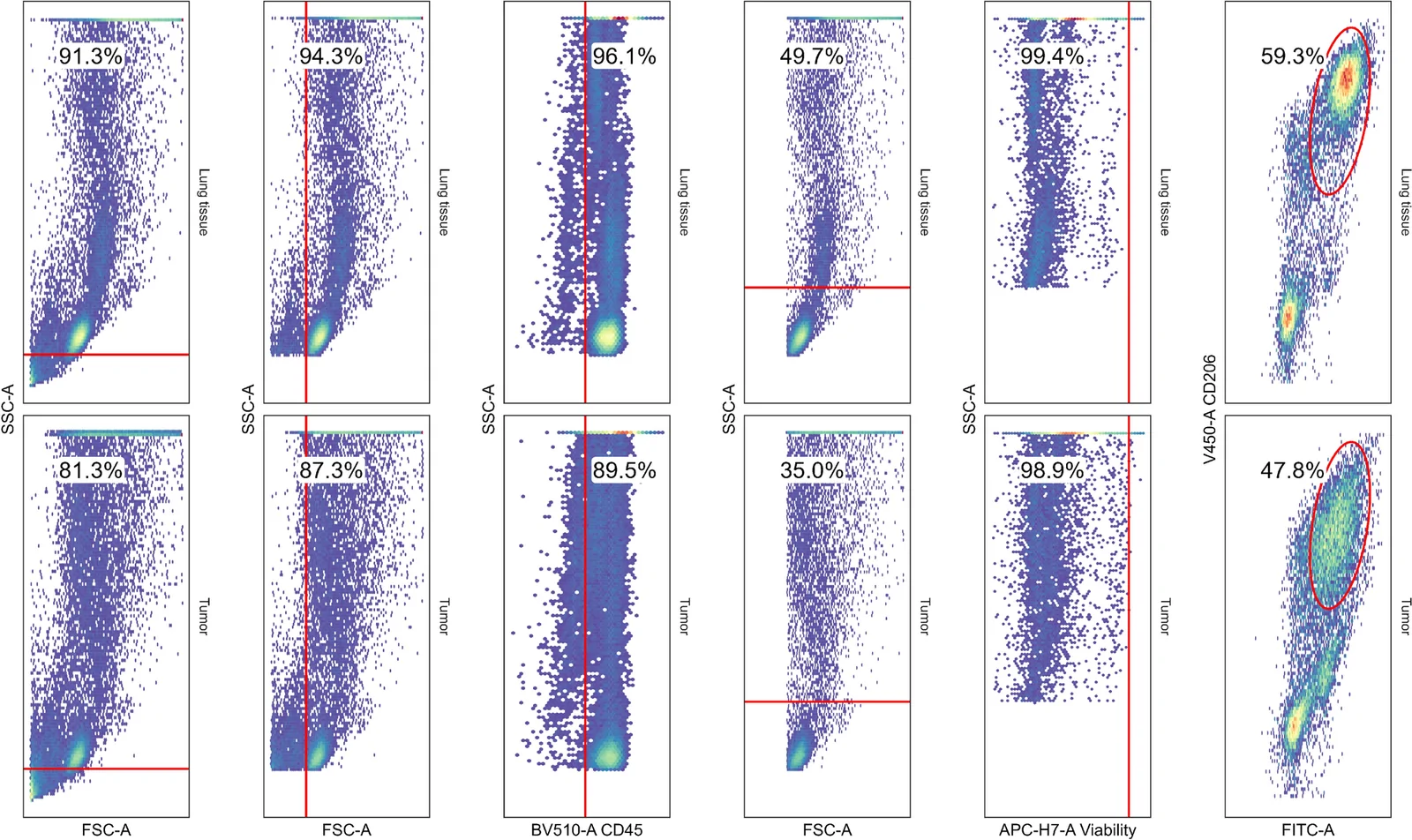
Gating strategy for FACS. After exclusion of lymphocytes (defined as viable CD45^+^ Side Scatter (SSc)^low^ and Forward Scatter (FSc)^low^ cells), macrophages were identified as viable, high autofluorescence (empty FITC channel^high^), CD45^bright^, SSc^high^, CD206^+^ cells

Immune cells isolated from tumor and lung tissue were stained for FACS, as previously described [21, 22]. The panel of antibodies for FACS are listed in supplementary Table 1. Cells were sorted using a BD FACS Aria III cell sorter (BD Biosciences, San Jose, CA, US) equipped with BD FACSDiva 8.0 software (BD Biosciences). The FACS gating strategy employed to isolate macrophages from lung tissue and tumor samples of a representative patient is shown in Fig. 1.

Post-sort purity of the macrophages from tumor and healthy lung tissue was assessed immediately after FACS and was on average 89.6% (Supplementary Fig. 1). The contaminating cells or debris was mostly CD45 low or dim, with lower autofluorescence compared to macrophages.

Sorted macrophages from tumor and lung tissue were collected in sterile polypropylene tubes (Corning) containing Roswell Park Memorial Institute medium 1640 (RPMI, Sigma-Aldrich Corp. St. Louis, US) supplemented with 30% fetal bovine serum and kept on ice. Cells were pelleted, lysed in 350 µl RLT buffer (Qiagen, Venlo, Netherlands), and stored at − 80 °C. RNA was isolated using the RNeasy Micro Kit (Qiagen) according to the manufacturer’s instructions. A yield of > 80 k macrophages were obtained from all samples.

### RNA extraction and sequencing

RNA sequencing data from TAMs and lung tissue macrophages were derived from the dataset utilized in a related study, and sequencing was performed following the procedures described therein [21]. The Genomics Core Facility (GCF), Department of Clinical and Molecular Medicine, Norwegian University of Science and Technology (NTNU) generated libraries for 22 samples, using the Lexogen SENSE mRNA-Seq library prep kit according to the manufacturer’s instructions (Lexogen GmbH, Vienna, Austria).

RNA sequencing libraries were quantified by qPCR using the KAPA Library Quantification Kit (Kapa Biosystems, Inc., Wilmington, MA, USA), pooled, and normalized to 2.6 pM. Libraries were clustered on two NextSeq 500 high-output flow cells. Single-read sequencing (75 cycles) was performed on a NextSeq 500 instrument (Illumina, Inc., San Diego, CA, USA) following the manufacturer’s protocol. FASTQ files were generated with bcl2fastq2 Conversion Software v2.17 (Illumina). Reads were filtered and trimmed using fastp v0.20.0, and transcript quantification was performed via quasi-alignment using Salmon v1.3.0 against the Ensembl GRCh38 release 92 transcriptome reference. Transcript-level estimates were imported into R (R Core Team, R Foundation for Statistical Computing, Vienna, Austria) and summarized to gene-level counts using the tximport package (v1.14.0, Bioconductor). Sequencing services were provided by the GCF, NTNU, which is funded by the Faculty of Medicine and Health Sciences at NTNU and the Central Norway Regional Health Authority.

### IHC

Tissue sections of 4 µm thickness from formalin fixed and paraffin embedded tumor tissue were mounted on glass slides. The slides were deparaffinized in xylene and rehydrated with ethanol. IHC analysis was performed using the Benchmark Ultra system (Ventana Medical Systems, Inc., Roche Group, Tucson, AZ, USA). The following antibodies and dilutions were used: CD4 mouse monoclonal antibody (clone SP35, dilution 1:100; catalogue number 104R-16; Cell MarqueTM, Rocklin CA, USA), CD8 mouse monoclonal antibody (clone L26, dilution 1:200; catalogue number M710301-2; Agilent Dako, Santa Clara, CA, USA), CD20 mouse monoclonal antibody (clone L26, dilution 1:200; catalogue number M075501-2; Agilent Dako, Santa Clara, CA, USA) and CD14 mouse monoclonal antibody (clone EPR 3653; ready to use, catalogue number 760–4523, Roche Diagnostics Corporation,Indianapolis, IN, USA). For antigen retrieval, the slides were incubated in CC1 for either 64 min (CD4, CD8 and CD14) or 16 min (CD20). Endogenous peroxidase activity was blocked with 3% hydrogen peroxide. Primary antibodies were applied, and the slides were then incubated at 37°C for either 32 min (CD4, CD8 and CD20) or 28 min (CD14).

Primary antibodies were visualized using the OptiView DAB IHC Detection Kit (for CD4, CD8 and CD20) and Ultraview DAB Detection Kit (for CD14), with 3,3′-diaminobenzidine (DAB) as the chromogen. Slides were counterstained with hematoxylin, dehydrated, cleared, and mounted using routine processing. For each antibody, control staining with an isotype-matched antibody was also performed.

### Digital IHC quantification

Stained IHC sections were scanned at × 40 resolution using a NanoZoomer S360 (Hamamatsu, Hamamatsu City, Japan). Tumor and adjacent parenchyma were evaluated on digitized whole-slide images using QuPath [24]. Tumor segmentation followed a three-step workflow based entirely in QuPath. First, nuclei and cells were segmented using the StarDist instance segmentation method via the QuPath StarDist extension [25]. Second, a supervised QuPath object classifier was trained on these StarDist-derived cell objects to distinguish tumor cells from non-tumor cells and other tissue elements. Third, QuPath’s density map functionality was applied to the classified tumor cells to generate continuous tumor annotations from regions with a sufficient density of tumor cell detections. Immune marker quantification was then performed within these tumor-derived regions. CD14 expression was quantified using a DAB pixel-thresholding classifier applied directly to the brown chromogen channel, whereas CD4, CD8, and CD20 were quantified at the cell level by measuring the nuclear DAB optical density (OD mean) within StarDist-detected cell objects classified as positive. All cell-density measurements were reported as positive cells per mm^2^ of annotated tumor area.

### Data analysis and statistical methods

The counts table from RNA sequencing was filtered for genes with low counts across samples, to avoid noise, as commonly recommended (we removed all genes with total counts sum < 100 across all samples), resulting in a counts table of approximately 21,400 genes in 22 samples (11 patients). Differential expression analysis of normalized gene counts was performed using the Bioconductor package DESeq2 (v1.50.0). DESeq2 fits a generalized linear model assuming a negative binomial distribution for the data and applies the Wald test to assess statistical significance. Details on the DESeq2 analysis and design formula are provided in the Supplementary Material.

Genes that were differentially expressed in TAMs from hot versus cold tumours were selected for further analysis with gene pathway- and gene ontology (GO) enrichment analyses (over representation analysis [26], using a hypergeometric test with the Benjamini–Hochberg method for false discovery rate control). GO enrichment analyses can be used to identify gene ontology terms that are over- or underrepresented in gene lists. The R/Bioconductor packages ReactomePA (v1.54.0) and clusterProfiler (v4.18.0) were used for gene pathway- and GO enrichment analyses.

Statistical analyses and data visualizations were conducted using R (version 4.5.1; R Core Team, R Foundation for Statistical Computing, Vienna, Austria) and Bioconductor (version 3.22), utilizing the tidyverse (v2.0.0) and ComplexHeatmap (v2.26.0) packages. Statistical comparisons were performed using Mann–Whitney test or Kruskal–Wallis tests as deemed appropriate. A *p*-value of < 0.05 was considered statistically significant.

## Results

### Classification of immune-hot and immune-cold NSCLC tumors

**Fig. 2:**
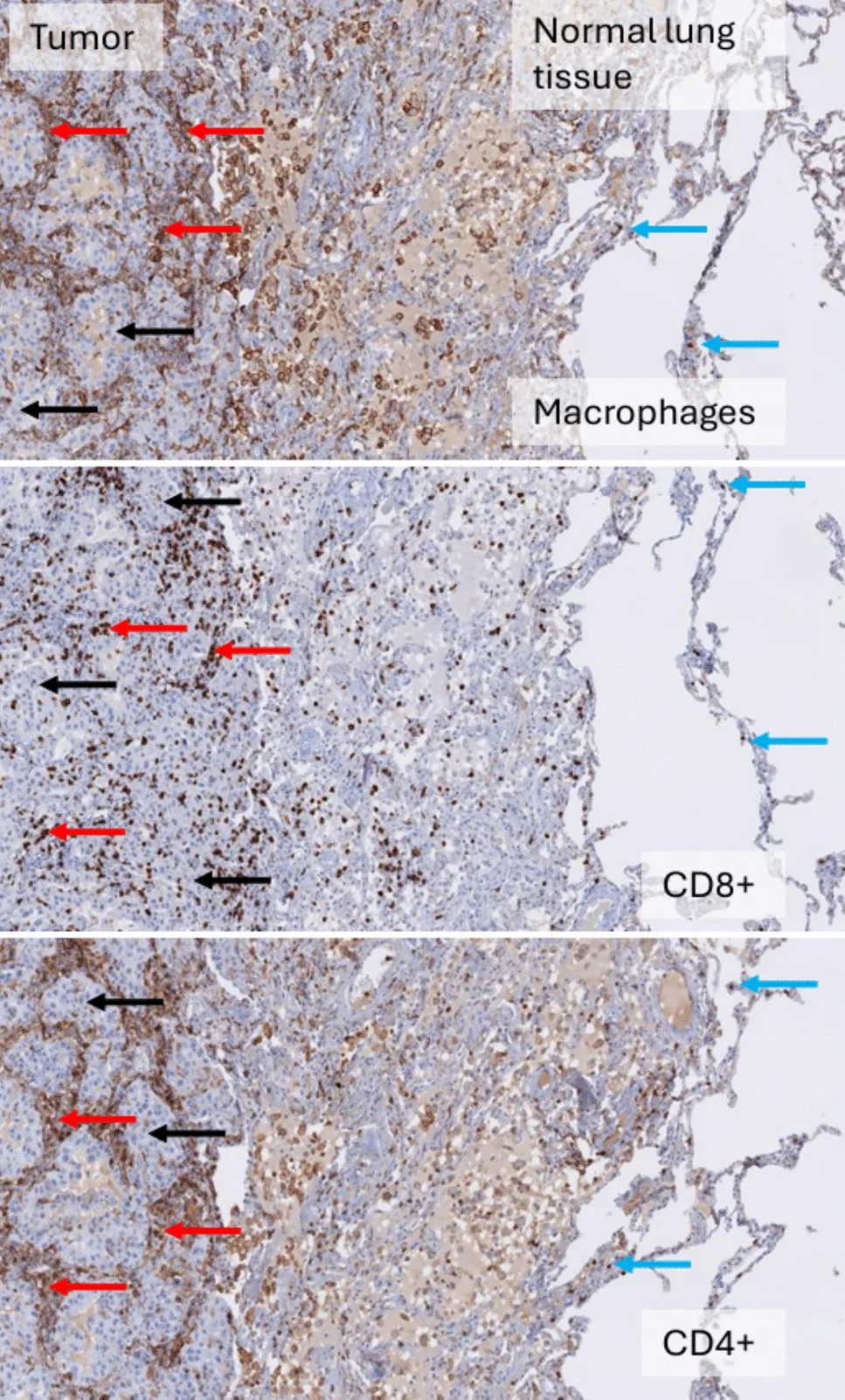
Immunohistochemical staining of immune cells in NSCLC tumor and adjacent normal lung tissue: Representative immunohistochemical staining of non–small cell lung cancer (NSCLC) tumor tissue and matched adjacent normal lung tissue sections showing CD14⁺ macrophages (upper panel), CD8⁺ T cells (middle panel), and CD4⁺ T cells (lower panel). Anti‑CD14, anti‑CD8, and anti‑CD4 antibodies were used to identify tumor‑associated macrophages (TAMs), CD8⁺ T cells, and CD4⁺ T cells, respectively. TAMs and tumor‑infiltrating lymphocytes (TILs) are predominantly located in close proximity to tumor cells, which can be identified by their characteristic morphological features. Red arrows indicate immune cells within tumor tissue; blue arrows indicate immune cells in adjacent normal lung tissue; black arrows indicate tumor cells

During surgery, tumor and adjacent healthy tissue was resected from 11 patients with clinical stage I-III NSCLC. Tissue sections were analysed via immunohistochemistry to identify TILs and TAMs. An example of IHC staining of immune cells in NSCLC tumor and adjacent normal lung tissue with anti-CD14, anti-CD4 and anti-CD8 to detect TAMs, CD4 + T cells and CD8 + T cells is shown in Fig. 2.

The densities of T cells (CD4 + and CD8 +), B cells (CD20 +), total TILs (CD4 + , CD8 + , CD20 +), and TAMs (CD14 +) were quantified (Table 1).

**Fig. 3:**
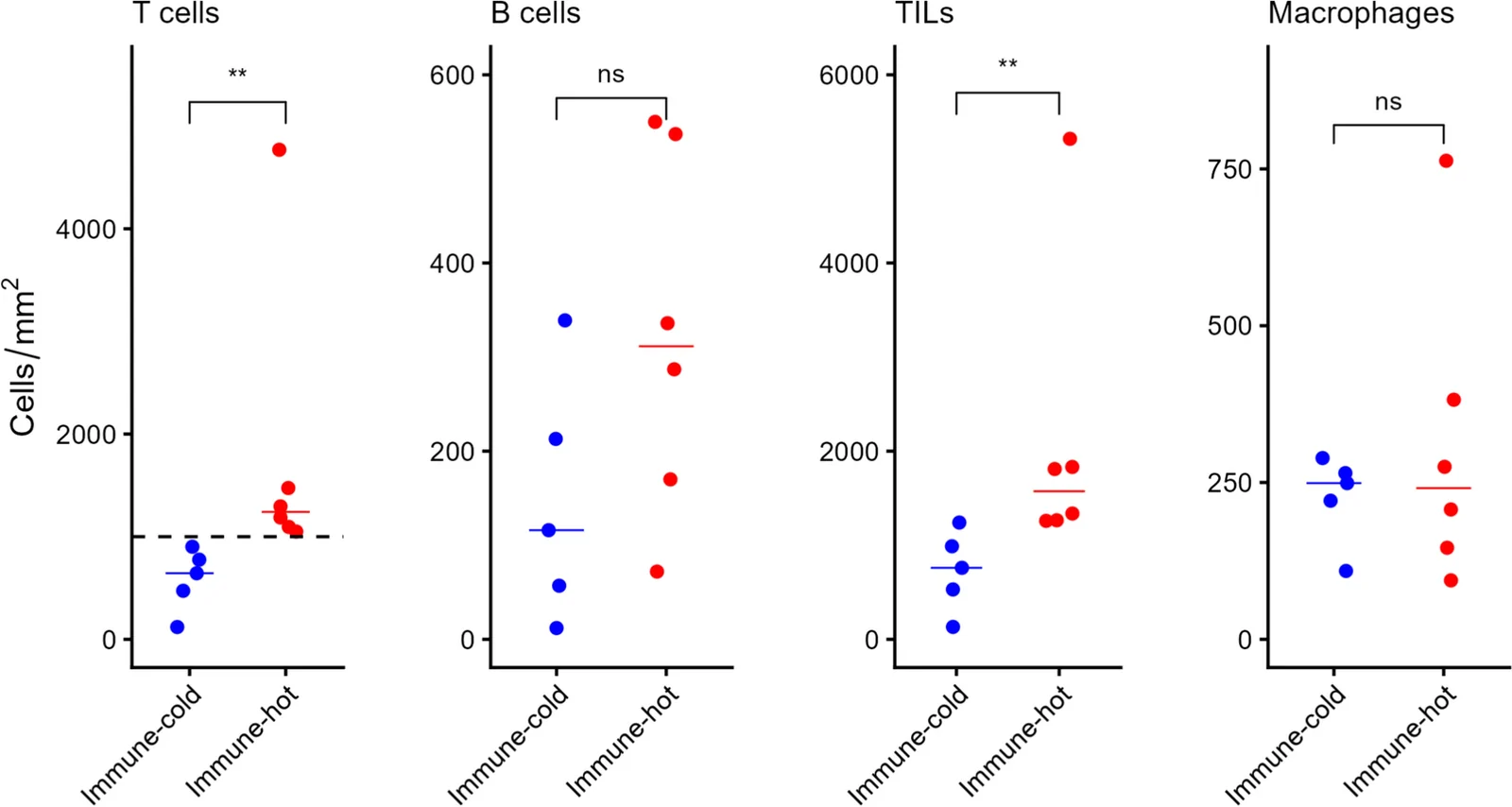
Density of T cells, B cells, TILs and TAMs in immune hot and cold tumors. Frequencies of T cells (CD4⁺, CD8⁺), B cells (CD20⁺), total TILs (CD4⁺, CD8⁺, CD20⁺), and TAMs (CD14⁺) in the TME were identified by digital IHC quantification. Immune‑hot tumors were defined by > 1000 T cells/mm^2^. Statistical comparisons were performed using the Mann–Whitney test. *: p < 0.05, **: p < 0.01, ns: not statistically significant; The median is marked with blue or red line

Although no universally accepted threshold exists to classify patients with immune-hot versus -cold tumors, a cut-off of > 1000 T cells /mm^2^ (CD4 + and CD8 +) was selected to identify immune-hot tumors, as this value has been utilized in previous studies [27]. Tumors from six patients were classified as immune-hot (T cell high), and tumors of five patients as immune-cold (T cell low). While the density of T cells (CD4 + and CD8 +) and total TILs (CD4 + , CD8 + and B cells) was significantly increased in immune-hot tumors, the densities of B cells and TAMs did not differ between immune-hot and -cold tumors (Fig. 3).

### Gene expression profiling in TAMs in cold versus hot NSCLC tumors reveals enrichment of specific gene sets

Portions of the resected tumor and adjacent healthy tissue were digested for immune cell isolation. TAMs and macrophages from healthy tissue were isolated by FACS prior to RNA extraction. Bulk RNA sequencing was then performed on macrophages from healthy tissue and on TAMs.

**Fig. 4:**
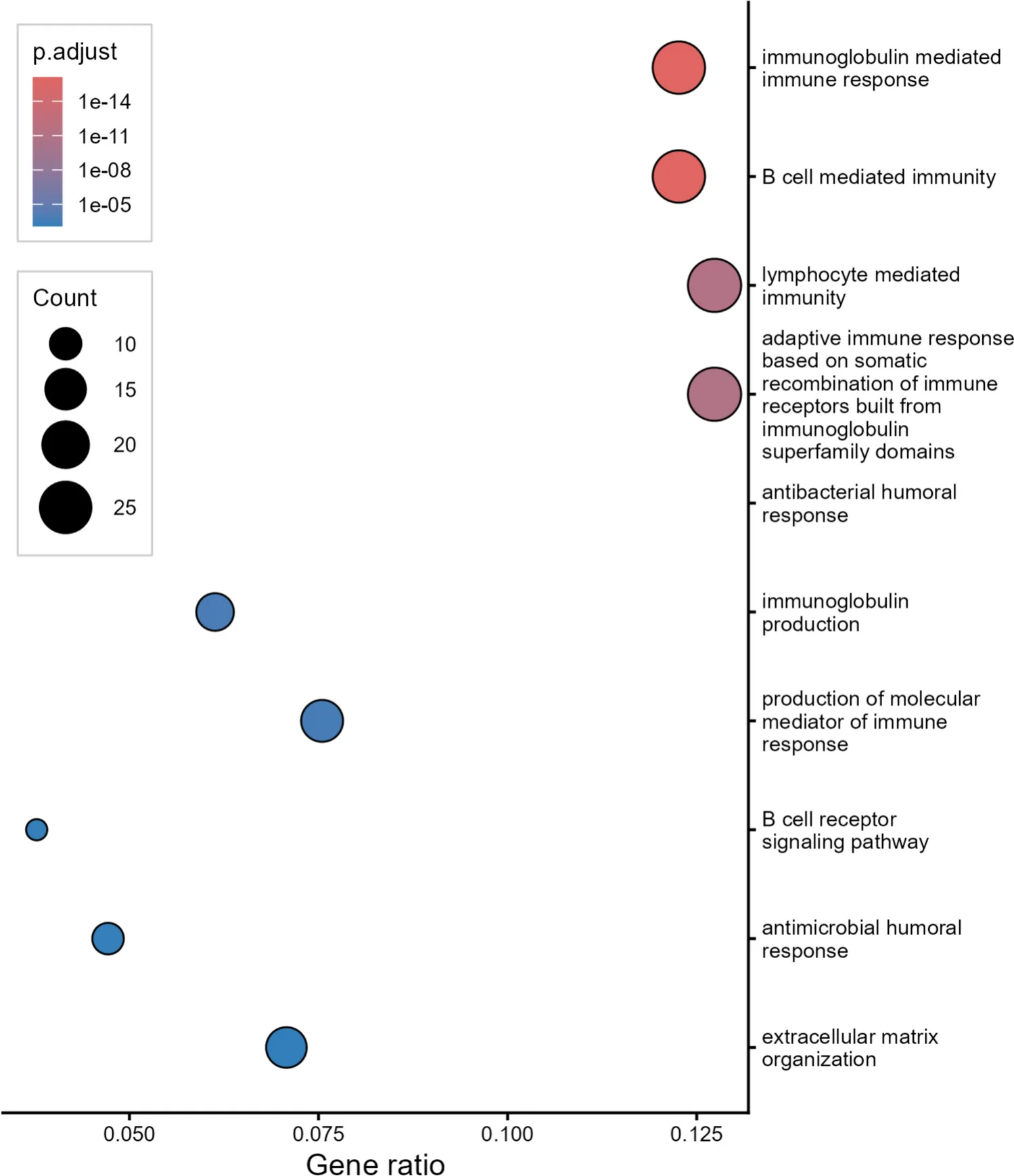
Gene Ontology (GO) enrichment analysis of differentially expressed genes (DEGs) in tumor‑associated macrophages (TAMs) from immune‑hot versus immune‑cold tumors. A total of 257 DEGs were identified and subjected to GO over‑representation analysis using a hypergeometric test, with p‑values adjusted for multiple testing using the Benjamini–Hochberg false discovery rate method. Enriched GO terms included pathways related to immunoglobulins, B‑cell–mediated immunity, and extracellular matrix organization. Gene Ratio represents the proportion of input DEGs annotated to a given GO term relative to the total number of DEGs included in the analysis

A total of 257 significantly differentially expressed genes (DEGs) were identified in TAMs from patients with immune-hot versus -cold tumors. Gene ontology over-representation analysis of these 257 genes indicated that several gene sets related to immunoglobulins were significantly enriched among the DEGs (Fig. 4). These included the gene sets ‘B cell mediated immunity’, ‘adaptive immune response based on somatic recombination of immune receptors built from immunoglobulin superfamily domains’ and ‘immunoglobulin production’. In addition, genes related to the ‘extracellular matrix organization’ were differentially expressed.

To explore whether the transcriptional differences observed between immune‑hot and immune‑cold tumors reflect broader trends in immune cell infiltration, we examined the relationships between expression of top differentially expressed genes from enriched pathways and intratumoral CD4⁺, CD8⁺, and total T‑cell densities quantified by IHC (Supplementary Figs. 2 and 3). Across samples, higher T‑cell densities were generally associated with lower expression of genes from both gene sets, with the most consistent inverse association observed for CD4⁺ T‑cell density. In contrast, associations with CD8⁺ T‑cell density were weaker and more variable (data not shown). Consistent with these observations, principal component analysis (PCA) of TAMs and lung tissue macrophage DEGs showed separation of immune‑hot and immune‑cold tumor macrophages along the first two principal components (Supplementary Fig. 4), suggesting that the applied T‑cell density threshold captures underlying transcriptional differences that align with variation in tumor immune contexture.

### Increased expression of genes related to immunoglobulins in TAMs from immune-cold NSCLC tumors

**Fig. 5:**
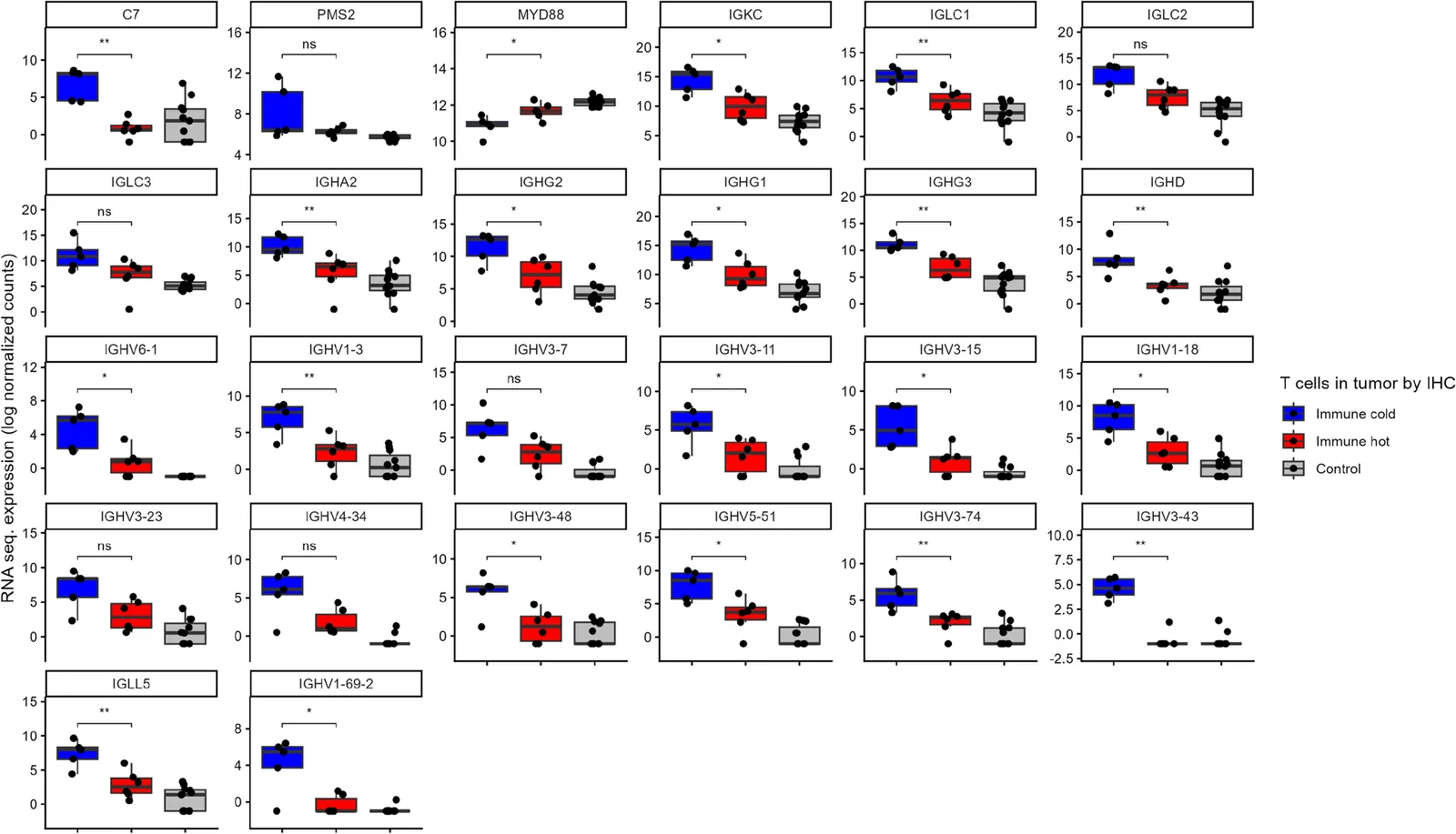
Expression of genes within the Gene Ontology pathway ‘'immunoglobulin mediated immune response’ in tumor-associated macrophages (TAMs) from immune-hot and immune-cold tumors. Normalized RNA expression levels of the 26 differentially expressed genes comprising this pathway are shown for TAMs from immune-cold and immune-hot tumors. Gene expression in macrophages from healthy lung tissue is included for contextual comparison. Statistical testing for visualization purposes was performed using the Kruskal–Wallis test; exact adjusted p-values from differential expression analyses are provided in Supplementary Table 2. *: *p* < 0.05, **: *p* < 0.01,* ns: not statistically significant.* Boxes indicate the interquartile range with the median shown; whiskers extend to 1.5 × the interquartile range, and points beyond this range are displayed as outliers. Expression values are shown as log2‑transformed DESeq2 size‑factor–normalized RNA counts

The top enriched gene set was ‘Immunoglobulin-mediated immune response’ (GO:0016064), comprising 212 genes; of these 26 were differentially expressed in TAMs from cold versus hot tumors (*p*-value: 8.1 × 10^–17^). Notably, 25 of the 26 identified differentially expressed immunoglobulin (Ig)-related genes exhibited increased expression in TAMs from immune-cold tumors compared to immune-hot tumors (Fig. 5). Effect sizes (log2 fold changes), adjusted *p*‑values, and DESeq2 dispersion estimates are reported for all DEGs in this gene set to contextualize both the magnitude of differential expression and the associated variability (Supplementary Table 2).

Macrophages from adjacent lung tissue displayed expression profiles for these genes similar to TAMs in immune-hot tumors. Notably, 23 of these DEGs were genes that are directly connected to IgG or IgA immunoglobulin protein expression. Genes coding for the Ig kappa and lambda light chains (IGKC, IGLC1, IGLC2, IGLC3) and the IgA and IgG heavy chains (IGHA2, IGHG1, IGHG2) were expressed at increased levels in TAMs from immune cold tumors. In addition, diversity (IGHD) and several variable (IGHV) gene segments were expressed at elevated levels. We also examined whether TAMs express genes required for somatic recombination of immunoglobulin loci, a process typically restricted to B cells. Transcription of RAG1, RAG2 and DNTT (TDT) were undetectable or near the detection limit in all TAM samples, while AICDA showed some expression. Thus, although TAMs from immune-cold NSCLC patients expressed genes encoding most components required for producing functional IgA or IgG molecules, they did not express genes required for V(D)J recombination. To rule out the possibility that immunoglobulin transcripts originated from contaminating B cells or plasma cells, we also assessed expression of canonical B cell markers. CD19, EBF1 and MS4A1 were absent or nearly absent in all samples, whereas CD22 and PAX5 showed low expression in some samples, but were neither significantly enriched in TAMs relative to macrophages from normal lung tissue nor differentially expressed between immune-cold and immune-hot tumors. CD27 and TNFRSF17 (BCMA) were absent or nearly absent in all samples. Expression of genes related to somatic recombination, canonical macrophage markers, and linage markers for T cells, B cells and plasma cells in macrophages from tumors and from healthy lung tissue, indicate no substantial contamination of B cells or plasma cells (Supplementary Fig. 5).

Other genes in this set overexpressed in TAMs from immune-cold tumors included Ig-lambda-like polypeptide 5 (IGLL5), which has been associated with B cell malignancies and renal cell carcinomas, the DNA repair gene PMS2 as well as the complement component C7. Notably, only the gene encoding the toll-like receptor signaling adaptor protein MyD88 (MYD88) exhibited reduced expression in TAMs from immune-cold tumors, potentially indicating dampened inflammatory signaling in this subset.

### Increased expression of extracellular matrix organization genes in TAMs from immune cold NSCLC tumors

**Fig. 6:**
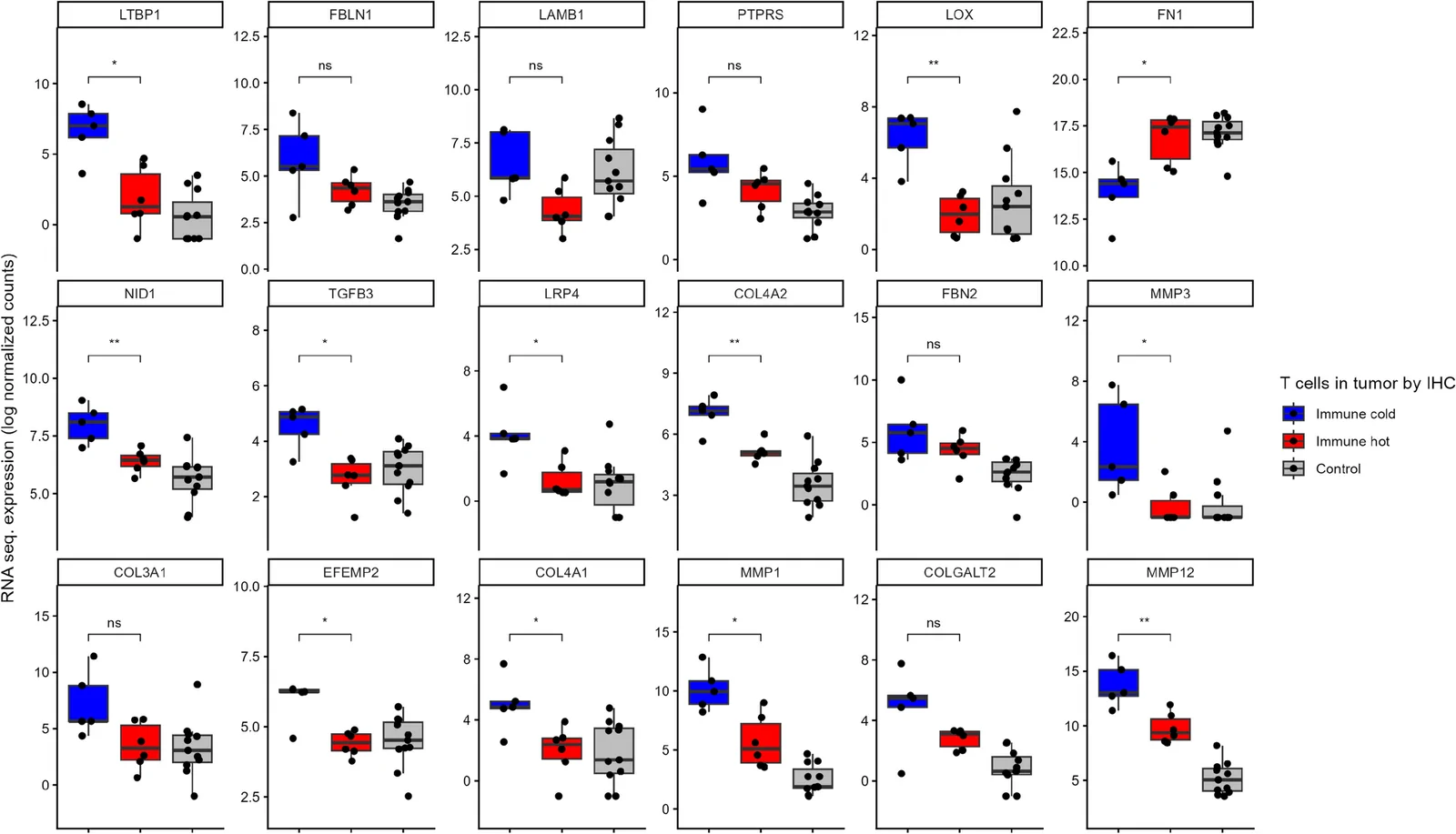
Expression of genes within the Gene Ontology pathway ‘extracellular matrix organization’ in tumor-associated macrophages (TAMs) from immune-hot and immune-cold tumors. Normalized RNA expression levels of the 18 differentially expressed genes comprising this pathway are shown for TAMs from immune-cold and immune-hot tumors. Gene expression in macrophages from healthy lung tissue is included for contextual comparison. Statistical testing for visualization purposes was performed using the Kruskal–Wallis test; exact adjusted p-values from differential expression analyses are provided in Supplementary Table 3. *: *p* < 0.05, **: *p* < 0.01, *ns: not statistically significant.* Boxes indicate the interquartile range with the median shown; whiskers extend to 1.5 × the interquartile range, and points beyond this range are displayed as outliers. Expression values are shown as log2‑transformed DESeq2 size‑factor–normalized RNA counts

The gene set ‘extracellular matrix organization’ (GO:0030198) was also highly enriched. 18 of 212 genes in this set were differentially regulated in TAMs from cold versus hot tumors (*p*-value: 8.1 × 10^–4^). Genes in this set influence a wide array of cellular functions including growth, migration, differentiation, survival, and immune responses. Macrophages in adjacent lung tissue mirrored TAM gene expression in immune‑hot tumors. Except for Fibronectin 1 (FN1), all DEGs in this set were upregulated in TAMs from immune-cold tumors (Fig. 6). FN1 was expressed at high levels in TAMs isolated from tumors as well as macrophages from healthy lung tissue, but with decreased levels in TAMs from immune-cold tumors. Matrix metalloproteases (MMP)-1, MMP-3, MMP-12, and the lysyl oxidase (LOX) gene were elevated in immune-cold compared to immune-hot tumors. Effect sizes (log2 fold changes), adjusted p‑values, and DESeq2 dispersion estimates are reported for all DEGs in this gene set to contextualize both the magnitude of differential expression and the associated variability (Supplementary Table 3). Variance estimation in the DESeq2 model was assessed by examining gene‑wise dispersion estimates plotted against mean normalized expression (Supplementary Fig. 6).

**Fig. 7:**
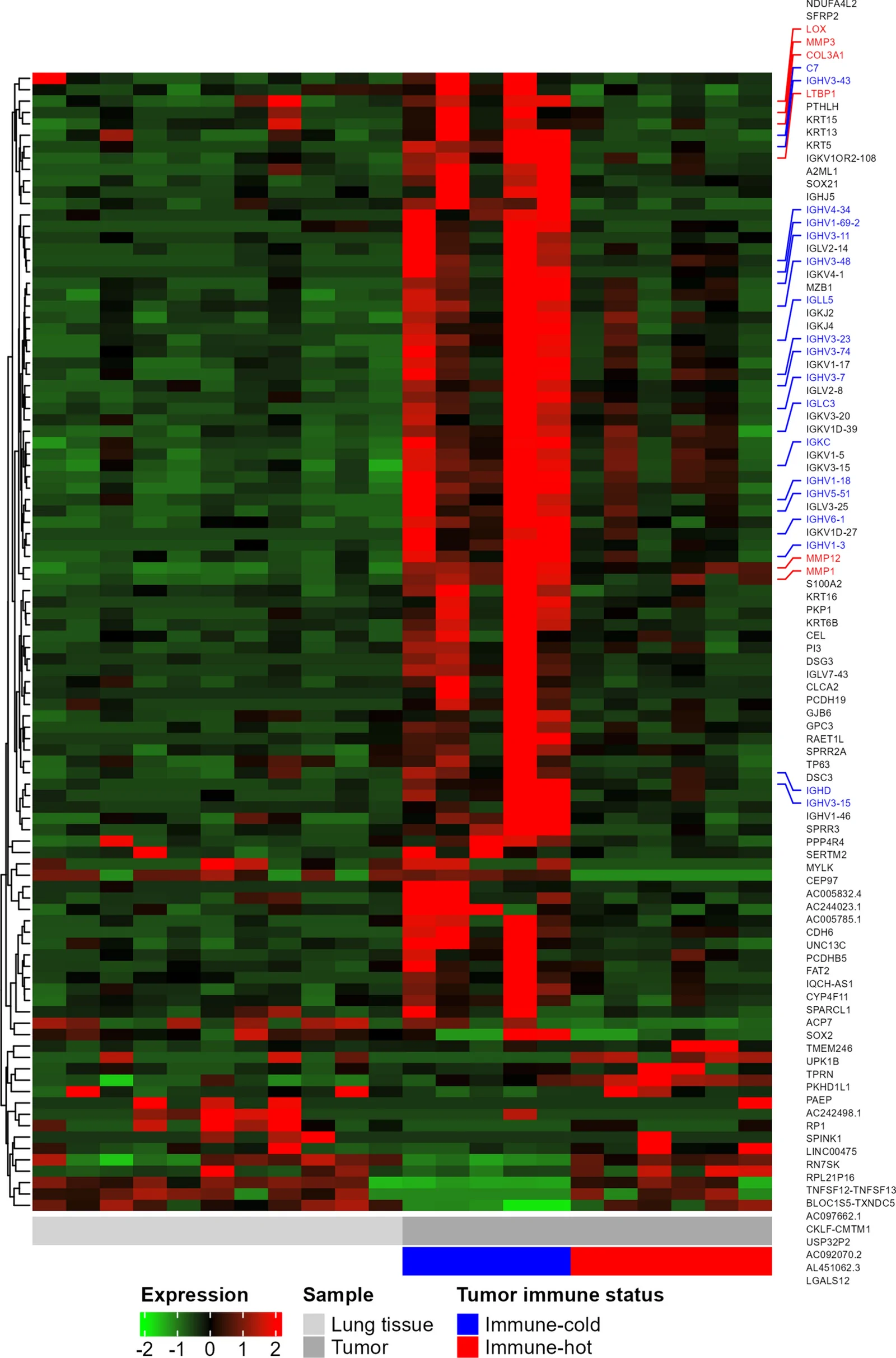
Expression profiles of top differentially expressed genes in tumor-associated macrophages from immune-hot versus immune-cold tumors. Heatmap displaying the relative expression levels of the top 100 differentially expressed genes in tumor‑associated macrophages isolated from immune‑hot and immune‑cold NSCLC tumors. Expression profiles from macrophages from adjacent healthy lung tissue are displayed for visualisation purposes (left side columns). Sidebar annotations highlight genes associated with the Gene Ontology terms ‘immunoglobulin‑mediated immune response’ (blue) and ‘extracellular matrix organization’ (red)

Comparison of the 100 top DEGs in TAMs from immune-hot versus immune-cold NSCLC tumors and macrophages from healthy tissue revealed that most of the identified DEGs were exclusively upregulated in TAMs originating from immune-cold tumors (Fig. 7). LGALS12 is one of few genes that were downregulated in immune-cold tumors. The human LGALS12 gene encodes galectin-12, an immune-related protein expressed in macrophages; loss of galectin-12 has been reported to promote M2 macrophage polarization [28].

## Discussion

In this study, we classified NSCLC tumors as either immune-hot or immune-cold and explored gene expression profiles in TAMs from the tumors. This approach enabled the identification of transcriptional differences that may define distinct macrophage functions in immune-hot and immune-cold tumors. Our results indicate that TAMs from immune-cold tumors express increased levels of numerous genes involved in immunoglobulin-mediated immune responses, whereas TAMs from immune-hot tumors lack this upregulation.

Immunoglobulins are thought to be primarily expressed in plasma cells and B cells as the principal components of the humoral immune response. However, immunoglobulin expression has been reported for a growing series of non-B linage cells, including normal lung epithelial cells and myeloid cells such as monocytes, macrophages, and granulocytes [29, 30]. According to one report, approximately 1–2% of circulating CD14^+^ monocytes in healthy individuals express antibodies [31]. Immunoglobulin expression in non-B cells was first documented in various cancer cells where secreted immunoglobulins appear to support tumor growth and survival [32]. Immunoglobulin gene rearrangement and IgG expression have been reported in cancer cells in NSCLC and have been linked to poor prognosis by promoting tumor immune escape and metastasis [33]. Expression of immunoglobulin-related genes in TAMs in various human tumors was also previously shown [34].

An alternative explanation for our findings is that TAMs acquire immunoglobulin mRNA through phagocytosis of B cells or plasma cells, or that incomplete purity during FACS contributes to the observed signal. However, the absence of mRNA for canonical B-cell or plasma-cell markers like CD19, EBF1, MS4A1 and CD27, and TNFRSF17, in macrophages with immunoglobulin production argues against contamination by mRNA of B cell or plasma-cell origin. Moreover, the restricted immunoglobulin repertoire reported for TAMs relative to B cells further supports that the immunoglobulin transcripts detected in TAMs are unlikely to originate from transferred B‑cell mRNA [34]. The precise mechanisms underlying immunoglobulin gene rearrangement and expression in TAMs remains to be determined, but expression of rearranged immunoglobulin genes in umbilical CD34^+^ progenitor cells [35] and in in vitro-generated myeloid colony-forming units [34] indicate that rearrangement of immunoglobulin genes occurs early in myeloid cell development. It was therefore not surprising that we did not find expression of RAG1, RAG2, and low expression of AICDA, genes involved in recombination of immunoglobulin genes, in this study of TAMs.

The function of immunoglobulin gene expression by TAMs in the TME is largely unknown, as is the role of the present finding of elevated immunoglobulin expression by TAMs in NSCLC tumors with lower concentration of TILs, immune-cold tumors. We do not have documentation for a causal relationship between immunoglobulin expression and lack of TILs in the TME, but the correlation between these phenomena gives an impetus to examine this topic. Some authors have proposed that immunoglobulin-expressing myeloid cells may represent a class of “smart phagocytes” capable of linking antigen-specific immunorecognition with innate effector functions [31].

In the present study, we further report differential expression of several genes related to extracellular matrix organization in cold versus hot tumors. Extracellular matrix MMPs are closely involved in tumor immunity, significantly affecting the innate immune response [36]. Remodeling of the extracellular matrix in carcinomas is associated with the establishment of an immunosuppressive environment [37]. MMPs and lysosomal enzymes secreted by TAMs have been linked to disrupting the integrity of the basement membrane and promoting metastasis and tumor cell migration in NSCLC [38]. COL4A1 and COL4A2 are potential regulators of tumor-supporting TME composition associated with poor prognosis [39]. COL4A1 and COL4A2 were upregulated in cold compared to hot tumors in the present study. The upregulation of genes involved in extracellular matrix organization in immune-cold tumors aligns with the current paradigm of TAM-induced immunosuppression, invasiveness, and immune escape in NSCLC. There is increasing evidence that TAMs comprise a spectrum of phenotypic states shaped by interactions within the tumor microenvironment. In particular, extracellular matrix‑educated TAMs display tissue‑remodeling and immunoregulatory features, are linked to impaired T‑cell function, and associate with immunosuppressive extracellular matrix programs and poor prognosis [37].

Fibronectin 1 (FN1) was significantly elevated in immune-hot tumors, although it was highly expressed in both immune-cold and -hot tumors. FN1 contains adhesion sites for integrins and mediates the interaction between cells and the extracellular matrix and is associated with tumor progression and M2 polarization in TAMs [40]. It is tempting to speculate that high production of FN1 by TAMs promotes anchoring of lymphocytes in the TME.

Several limitations should be considered when interpreting the findings of this study. First, Using bulk RNA sequencing, subsets of TAMs or gene expression at the single-cell level could not be discerned. As a result, differences between our findings and previously published TAM expression profiles may reflect spatial and temporal heterogeneity within the TME [41].

Second, although the cohort size (*n* = 11) is reasonable for a surgically based study, it limits statistical power, particularly for genome‑wide differential expression analyses, and reduces the stability of variance estimates, leading to increased uncertainty in gene‑wise dispersion estimation [42]. While DESeq2 applies dispersion shrinkage to stabilize variance estimates and reduce false‑positive findings, the identification of a relatively large number of DEGs should nonetheless be interpreted with caution, especially in analyses of tumors and immune cell populations, which are known to exhibit substantial inter‑individual heterogeneity. To address this limitation, we emphasized consistent expression patterns and pathway-level enrichment rather than reliance on individual genes in isolation. Notably, genes associated with immunoglobulin-mediated immune responses and extracellular matrix regulation showed robust effect sizes and concordant regulation across samples derived from immune-cold tumors, supporting the biological relevance of these findings despite limited sample size.

Third, classification of tumors as immune-hot or immune-cold required dichotomization of a biologically continuous variable. While no universally accepted cutoff for intratumoral T-cell density exists, the selected threshold was informed by prior literature and cohort-specific distributions. Importantly, sensitivity analysis using an alternative cutoff, resulting in reassignment of one sample, preserved the principal transcriptional findings, indicating that the observed TAM signatures are not artifacts of the specific threshold chosen. Nonetheless, future studies with larger cohorts could benefit from modeling immune infiltration as a continuous variable to more fully capture biological gradients within the TME.

An additional methodological limitation relates to the identification and isolation of macrophages. TAMs were defined based on CD45 brightness, autofluorescence, scatter characteristics, and CD206 expression. Although CD206 is ubiquitously expressed by lung macrophages [43–45] and is characteristic of M2‑polarized TAMs in NSCLC [45], its expression is not restricted to macrophages and may also be detected on subsets of monocytes and immature dendritic cells. Autofluorescence was included as an additional discriminating feature, as it is typically high in lung macrophages, but overlap with other myeloid populations cannot be entirely excluded. Accordingly, a low level of contamination by other myeloid cells cannot be ruled out. However, dendritic cells generally constitute a minor fraction of the myeloid compartment in both tumor and normal lung tissue, and post-sort purity assessments demonstrated comparable levels of non-macrophage events across corresponding samples, suggesting that such contamination is unlikely to substantially influence the observed transcriptional patterns. An additional limitation of this study is the lack of independent validation of the RNA sequencing results at the mRNA or protein level, which will be important to confirm the observed transcriptional differences in future studies. Future studies incorporating larger patient cohorts, refined immunophenotyping, single-cell and spatial transcriptomic technologies will be important to validate and extend these findings and to further clarify their clinical relevance.

In conclusion, bulk RNA-sequencing of FACS-sorted immune cells from NSCLC demonstrates the upregulation of genes related to immunoglobulins and immunoglobulin-mediated immune responses in TAMs from immune-cold compared to immune-hot tumors. Additionally, genes involved in extracellular matrix organization were differentially expressed in TAMs from cold versus hot tumors. Though we cannot yet conclude what the observed activation of these gene pathways in TAMs from immune-cold tumors mean functionally for the TME, our results highlight the possibility that TAM function may be profoundly altered in immune-cold versus -hot tumors, and that these differences may contribute to regulatory processes that ultimately restrict T cell infiltration to immune-cold tumors. To determine whether TAMs exert distinct regulatory functions within the TME of immune‑hot versus immune‑cold NSCLC tumors, future studies should incorporate high‑resolution transcriptomic profiling of TAMs, tumor cells, and immune‑cell subsets in larger, well‑characterized patient cohorts. As TAMs represent key regulators of the tumor microenvironment that influence the ability of the immune system to control or kill cancer cells [14], our findings may have important implications for the design of future TAM-directed immunotherapies.

## Supplementary Information

Below is the link to the electronic supplementary material.

Supplementary file1 (DOCX 4703 KB)

## Data Availability

We will be happy to make all research data available with this article. The RNA data files and metadata are quite large files and are not easily uploaded during the submission process.

## Abbreviations

AC: Adenocarcinoma
DAB: 3,3′-Diaminobenzidine
DEGs: Differentially expressed genes
FACS: Fluorescence-activated cell sorting
FSc: Forward scatter
GO: Gene ontology
IHC: Immunohistochemistry
MHC: Major histocompatibility complex
MMP: Matrix metalloproteases
NSCLC: Non-small cell lung cancer
PD-1 (pathway): Programmed death 1 (pathway)
PD-L1: Programmed death-ligand 1
SSc: Side scatter
SCC: Squamous cell carcinoma
TAMs: Tumor-associated macrophages
TILs: Tumor-infiltrating lymphocytes
TME: Tumor microenvironment
VATS: Video-assisted thoracoscopic surgery
