# Supplementary material for: Immune-cold NSCLC tumors harbor tumor-associated macrophages with elevated expression of immunoglobulin genes
Source: Cancer Immunol Immunother. 2026 Jun 6;75(9):208. doi: 10.1007/s00262-026-04446-4 (PMC13490338; doi:10.1007/s00262-026-04446-4)
Supplement: Supplementary file 1 [file 262_2026_4446_MOESM1_ESM.docx]

**Supplementary materials:**

**Cancer Immunology, Immunotherapy (submitted in 2026) – Markus Haug et al.**

**Supplementary tables:**

**Marker**	**Fluorochrome**	**Supplier**

Fixable Viability dye	eFluor780	eBioscience_1_

CD45	Brilliant Violet 510	BD Biosciences_2_

CD206	Brilliant Violet 421	BD Biosciences_2_

CD4	PE-CF594	BD Biosciences_2_

CD8	PerCP/Cyanine5.5	BD Biosciences_2_

**Supplementary table 1: Panels of antibodies for fluorescence-activated cell sorting (FACS).**

_1_ eBioscience, San Diego, CA, USA; _2_ BD Biosciences, San Jose, CA, US

| **gene_name** | **log2FoldChange** | **lfcSE** | **baseMean** | **padj** | **dispMAP** | **dispGeneEst_over_dispFit** |
| --- | --- | --- | --- | --- | --- | --- |
| C7 | -6,81457871 | 1,28497782 | 55,6843803 | 0,00011279 | 1,90155203 | 7,366714606 |
| PMS2 | -3,56823838 | 0,6792996 | 258,979622 | 0,00014071 | 0,59798383 | 3,247851933 |
| IGHV3-43 | -6,29234937 | 1,21896082 | 6,82001185 | 0,0002076 | 1,0182972 | 0,613351387 |
| IGHV3-74 | -4,80473388 | 0,98005303 | 31,3334418 | 0,00055176 | 1,15153841 | 3,186244842 |
| IGHD | -6,41902326 | 1,36115891 | 379,884159 | 0,0010615 | 2,02883967 | 12,5923312 |
| IGKC | -4,19228947 | 0,8942365 | 10395,0092 | 0,00117176 | 1,04759874 | 6,927817384 |
| IGLC3 | -4,70762906 | 1,01704457 | 2507,83538 | 0,00139719 | 1,35413837 | 8,937827391 |
| IGHV3-15 | -5,13194162 | 1,1944558 | 28,0797501 | 0,00430212 | 1,72490926 | 4,860236221 |
| IGLC1 | -3,75961911 | 0,90387965 | 612,176436 | 0,00637731 | 1,0676239 | 6,690815668 |
| IGHG2 | -3,8809007 | 0,94169975 | 1284,45811 | 0,00704487 | 1,16052117 | 7,476004844 |
| IGLL5 | -4,23747362 | 1,03166106 | 73,0509175 | 0,00728693 | 1,36255038 | 5,918337668 |
| IGHG3 | -3,84485601 | 0,95256671 | 798,277253 | 0,00940939 | 1,18664463 | 7,600615764 |
| IGHV1-69-2 | -6,34248459 | 1,60203066 | 9,52766191 | 0,01243734 | 2,48139558 | 3,838007476 |
| IGHV1-3 | -4,3900748 | 1,12608934 | 54,1364342 | 0,01433761 | 1,61379601 | 6,276926441 |
| IGHV4-34 | -4,42723038 | 1,13867199 | 30,807111 | 0,01466607 | 1,61470291 | 4,70728048 |
| IGLC2 | -3,89599481 | 1,00937567 | 1689,10704 | 0,01537766 | 1,33388784 | 8,844868567 |
| IGHV1-18 | -5,07066424 | 1,31480534 | 143,826596 | 0,01537766 | 1,84510584 | 9,962570001 |
| IGHA2 | -3,85644414 | 1,01538706 | 502,582603 | 0,0180043 | 1,34719576 | 8,495808331 |
| IGHV3-48 | -4,50206733 | 1,19884195 | 25,623675 | 0,01993348 | 1,77464015 | 4,582426448 |
| IGHV5-51 | -4,18846873 | 1,14606268 | 109,899705 | 0,02553633 | 1,70058898 | 9,037390118 |
| IGHV3-11 | -4,19063151 | 1,18654941 | 26,506573 | 0,03458957 | 1,75850735 | 4,800850766 |
| MYD88 | 0,80102992 | 0,23322179 | 3700,97548 | 0,04376466 | 0,07083737 | 0,350140956 |
| IGHV3-23 | -3,98813418 | 1,16701563 | 71,7049677 | 0,04569004 | 1,75639869 | 7,621798342 |
| IGHV6-1 | -4,53213481 | 1,33335192 | 13,5337908 | 0,04789442 | 2,12596615 | 3,789449864 |
| IGHG1 | -3,87929993 | 1,14353073 | 11303,2964 | 0,04860459 | 1,3514652 | 10,02882661 |
| IGHV3-7 | -4,80003901 | 1,41504287 | 75,7856845 | 0,04860459 | 1,95971947 | 9,434018959 |

**Supplementary table 2: Effect sizes (log2 fold changes) and dispersion‑related metrics for all DEGs in the 'immunoglobulin mediated immune response’ gene set in tumor‑associated macrophages from immune-hot and immune-cold tumors.** Differential expression analysis was performed using DESeq2. Effect sizes are reported as shrunken log2 fold changes estimated using the apeglm method to stabilize estimates in the context of limited sample size. Gene‑wise dispersion was estimated using DESeq2’s shrinkage framework and used for Wald significance testing.

| **gene_name** | **log2FoldChange** | **lfcSE** | **baseMean** | **padj** | **dispMAP** | **dispGeneEst_over_dispFit** |
| --- | --- | --- | --- | --- | --- | --- |
| MMP12 | -4,42829489 | 0,80926329 | 6852,29988 | 5,2948E-05 | 0,85776451 | 5,61908907 |
| FN1 | 2,75734149 | 0,55894182 | 128927,559 | 0,00049802 | 0,40933902 | 1,84300849 |
| MMP1 | -4,00224395 | 0,87455658 | 544,297283 | 0,00168939 | 0,99850829 | 6,12074482 |
| LOX | -4,59850346 | 1,11438163 | 39,0178901 | 0,00699367 | 1,51312647 | 4,51768015 |
| COLGALT2 | -3,2798191 | 0,83221767 | 18,2066812 | 0,01316325 | 0,83108863 | 1,44148965 |
| LAMB1 | -2,44847446 | 0,62974869 | 96,8858441 | 0,01466607 | 0,49636788 | 2,09780205 |
| TGFB3 | -1,95472271 | 0,50413423 | 11,7023679 | 0,0150767 | 0,2363151 | 0,21149236 |
| COL3A1 | -4,89063523 | 1,28606518 | 183,996076 | 0,01798424 | 1,85622524 | 10,0843454 |
| NID1 | -1,71933955 | 0,45881235 | 120,442305 | 0,02027105 | 0,26851964 | 1,17525695 |
| FBLN1 | -2,45337948 | 0,66577169 | 37,6293667 | 0,02373171 | 0,55288891 | 1,51288174 |
| LTBP1 | -4,33158091 | 1,17924825 | 40,5847562 | 0,02429479 | 1,76034042 | 5,90882614 |
| EFEMP2 | -1,63936012 | 0,44801868 | 33,659898 | 0,02524952 | 0,2323113 | 0,53663891 |
| FBN2 | -3,19030381 | 0,8827929 | 65,0008938 | 0,0277537 | 1,00076373 | 3,81452446 |
| LRP4 | -3,71412625 | 1,0400004 | 10,5578725 | 0,03139031 | 1,22371346 | 1,38708481 |
| COL4A2 | -1,97594443 | 0,55401939 | 50,5313619 | 0,03155469 | 0,38530431 | 1,20689358 |
| MMP3 | -6,41579647 | 1,80398441 | 15,6101646 | 0,03225582 | 3,60513368 | 7,2249416 |
| COL4A1 | -3,4740659 | 0,98120645 | 18,723614 | 0,03377001 | 1,16502554 | 2,137667 |
| PTPRS | -2,65456062 | 0,76359128 | 41,1993418 | 0,04049206 | 0,73805019 | 2,17847051 |

**Supplementary table 3: Effect sizes (log2 fold changes) and dispersion‑related metrics for all DEGs in the 'extracellular matrix organization’ gene set in tumor‑associated macrophages from immune-hot and immune-cold tumors.** Differential expression analysis was performed using DESeq2. Effect sizes are reported as shrunken log2 fold changes estimated using the apeglm method to stabilize estimates in the context of limited sample size. Gene‑wise dispersion was estimated using DESeq2’s shrinkage framework and used for Wald significance testing.

**Supplementary figures:**

**Supplementary figure 1: Post-sort purity of macrophages from tumor and lung tissue**. The average post-sort purity of macrophages from lung tissue (upper panel) and tumor (lower panel) was 89.6 % (91.5 % in healthy lung tissue and 87.6 % in tumors). The contaminating cells or debris was mostly CD45 low or dim, with lower autofluorescence compared to macrophages (CD45 not shown in the present plot), and the phenotypic pattern (including CD45, side- or forward scatter properties) of contaminating cells was similar in samples from tumors and healthy lung tissue from the individual patients.

**Supplementary figure 2: Association between expression of genes in the immunoglobulin mediated immune response gene set in tumor associated macrophages (TAMs) and tumor T cell density.** Normalized relative RNA expression levels of the 26 differentially expressed genes belonging to the Gene Ontology pathway *immunoglobulin‑mediated immune response* in TAMs isolated from immune-hot and immune-cold NSCLC tumors are plotted against T cell densities quantified by immunohistochemistry (IHC; cells/mm²). The IHC-based cutoff used to classify tumors as immune-hot or immune-cold (1000 T cells/mm²) is indicated to illustrate the dichotomization of samples.

**Supplementary figure 3: Association between expression of genes in the immunoglobulin mediated immune response gene set in tumor associated macrophages (TAMs) and tumor CD4+ T cell density.** Normalized relative RNA expression levels of the 26 differentially expressed genes belonging to the Gene Ontology pathway immunoglobulin mediated immune response in TAMs isolated from immune-hot and immune-cold NSCLC tumors are plotted against CD4+ lymphocytes (mainly T cells) densities quantified by immunohistochemistry (IHC; cells/mm²). The IHC-based cutoff used to classify tumors as immune-hot or immune-cold (1000 T cells/mm²) is indicated to illustrate the dichotomization of samples.

**Supplementary figure 4: Principal component analysis (PCA) of differentially expressed genes (DEGs) in macrophages from immune-hot and immune-cold tumors.** PCA of DEG expression profiles shows a clear separation between macrophages derived from tumor tissue and those from normal lung tissue. Macrophages from immune-hot tumors cluster separately from immune-cold tumor macrophages, with generally higher scores along principal component 1 (PC1), and lower scores along principal component 2 (PC2). Macrophages from healthy lung tissue are included as controls. T cell densities assessed by immunohistochemistry (IHC; cells/mm²) are displayed as sample labels for tumor-derived macrophages to illustrate inter-sample heterogeneity.

**Supplementary figure 5: Expression of lineage markers and somatic recombination–related genes in macrophages from tumor and normal lung tissue.** Gene expression of canonical lineage markers for macrophages, T cells, B cells, and plasma cells, as well as genes associated with somatic recombination, was assessed in tumor-associated macrophages (TAMs) and in macrophages isolated from healthy lung tissue. Both TAMs and macrophages from normal lung exhibited high expression of established macrophage markers, whereas canonical T cell, B cell, and plasma cell markers were absent or expressed at low levels across all samples. Moderate expression of CD4, CD22, and CD38, as well as the plasma cell–associated marker SDC1 (CD138), was observed and is consistent with reported expression of these molecules in macrophages under certain physiological or pathological conditions. In contrast, TNFRSF17 (BCMA), another plasma cell marker, was nearly undetectable. Genes involved in somatic recombination, including those associated with V(D)J recombination, were absent or expressed at near‑background levels, indicating a lack of active recombination processes in macrophages. APEX1 showed moderate to high expression, consistent with its known roles in DNA repair and cellular stress responses in macrophages. The immunoglobulin kappa constant gene (IGKC) was included for comparative purposes and was highly expressed in TAMs from immune‑cold tumors, in contrast to its low expression in macrophages from normal lung tissue.

**Supplementary figure 6: Dispersion estimates from the DESeq2 model.**

Gene‑wise dispersion estimates (maximum a posteriori, MAP) are plotted against mean normalized expression (baseMean) for all genes. Dispersions were estimated using DESeq2 and represent the final moderated variance estimates used for differential expression testing. Axes are shown on a logarithmic scale.

**Supplementary information:**

**Details on the DESeq2 analysis and design formula:**

The DESeq2 analysis was performed using the following essential steps and design specification:

**counts <- round(counts) %>%**

**as.data.frame() %>%**

**dplyr::filter(rowSums(.) >= 100)**

**de <- DESeqDataSetFromMatrix(**

**countData = counts,**

**colData = sample.info,**

**design = ~ t_high**

**)**

**de <- DESeq(de, betaPrior = FALSE)**

**res <- results(de, name = "t_high_cancer_yes_vs_cancer_no", tidy=TRUE)**

The R object counts contains the preprocessed raw count matrix. Prior to analysis, genes with a total count sum across all samples <100 were filtered out, a commonly applied procedure to reduce noise in RNA‑seq count data before DESeq2 analysis.

The sample.info object is a data frame containing sample annotations, including immunohistochemistry‑derived immune cell densities and tissue type (tumor or lung tissue). The design formula includes the variable t_high, a four‑level factor encoding immune status (immune‑hot vs immune‑cold) and tissue type (tumor vs lung). The contrast *t_high_cancer_yes_vs_cancer_no* refers to the differential expression analysis comparing immune‑hot and immune‑cold tumor samples.
